# Does Personality Change Follow Deep Brain Stimulation in Parkinson's Disease Patients?

**DOI:** 10.3389/fpsyg.2021.643277

**Published:** 2021-07-30

**Authors:** Joshua A. Wilt, Amanda R. Merner, Jaclyn Zeigler, Michelle Montpetite, Cynthia S. Kubu

**Affiliations:** ^1^Department of Psychological Sciences, Case Western Reserve University, Cleveland, OH, United States; ^2^Department of Neurology, Cleveland Clinic, Cleveland, OH, United States; ^3^Cleveland Clinic Lerner College of Medicine of Case Western Reserve University, Cleveland, OH, United States

**Keywords:** personality, deep brain stimulation, personality change, Parkinson's disease, personality pathology

## Abstract

Deep Brain Stimulation (DBS) has emerged as a safe, effective, and appealing treatment for Parkinson's Disease (PD), particularly for improving motor symptoms (e. g., tremor, bradykinesia, and rigidity). However, concerns have been raised about whether DBS causes psychological changes, including changes to personality: characteristic and relatively stable patterns of affect, behavior, cognition, and desire. In this article, after first presenting some background information about PD and DBS, we examined evidence obtained from various empirical research methods (quantitative, qualitative, and mixed methods for evaluating patient valued characteristics) pertaining to whether DBS causes personality change. General limitations across research methods include a lack of randomized clinical trials and small sample sizes. We organized our review of findings according to different layers of personality variables: dispositional traits (including personality pathology), characteristic adaptations, and narrative identity. Though most work has been done on dispositional traits, there is not much evidence that dispositional traits change following DBS. Little work has been done on characteristic adaptations, but there is somewhat consistent evidence for positive perceived progress toward goals across a number of domains: routine activities, work, social/relational, and leisure. Nascent work on narrative identity holds promise for revealing issues around self-image that may be common following DBS. We listed a number of strategies for advancing research, highlighting opportunities related to personality conceptualization, personality assessment, and interdisciplinary scholarship. Finally, we offer practical applications of our findings for the informed consent process and for ongoing treatment.

## Does Personality Change Follow Deep Brain Stimulation in Parkinson's Disease Patients?

Parkinson's disease (PD) is a progressive neurodegenerative disorder affecting ~3 per 1,000 people over age 40 (Pringsheim et al., 2014). Classic symptoms of PD include tremor, bradykinesia, rigidity, and postural instability (Jankovic, 2008). There has been growing recognition of the importance of non-motor symptoms in PD (which vary in prevalence across different stages of the disease), including sensory processing difficulties, sleep problems, pain, autonomic changes, anxiety, depression, diminished initiative, psychotic symptoms, and dementia (Pandya et al., 2008; Löhle et al., 2009). The primary classic morphological indicator of PD is the loss of pigmented neurons in the substantia nigra pars compacta reflecting loss of dopaminergic neurons; however, neuropathological studies have documented that the disease involves multiple brain regions and neurotransmitter systems as it progresses (Braak et al., 2006; Dickson, 2012) consistent with the diverse symptoms associated with the disease.

First line treatments for PD involve dopaminergic medications (i.e., levodopa or dopamine agonists) to address motor symptoms (Rogers et al., 2017). If medication does not control motor symptoms or produces intolerable side effects, deep brain stimulation (DBS) may be considered as a treatment option. The most common site for DBS in the treatment of motor symptoms of PD is the subthalamic nucleus to address most cardinal motor symptoms of PD as well as reduce medication burden, although the ventral intermediate nucleus of the thalamus (to address tremor-predominant PD) or globus pallidus interna (when patients are highly susceptible to dyskinesias) may also be considered depending on the specific symptom profile, team preferences, and other factors (Rezai et al., 2008; Groiss et al., 2009). DBS entails surgical implantation of electrodes to deliver stimulation to specific brain regions. The electrodes are connected to a neuropacemaker that can be programmed by a clinician to deliver electrical stimulation at different amplitudes, pulse width (i.e., average power), and frequency. DBS has several advantages over previous ablative neurosurgical treatments for PD. First, it is reversible (i.e., the entire system can be explanted) and provides the treatment team the opportunity to maximize benefit while minimizing any potential side-effects *via* virtually unlimited programming options (Rezai et al., 2008). Finally, the patient is able to turn the DBS system on and off (Pugh, 2019). The safety and efficacy of DBS in treating many motor symptoms of PD has been well-demonstrated [see Deuschl et al. (2006b), Rezai et al. (2008), Weaver et al. (2009), and Limousin and Foltynie (2019)] with relatively mild cognitive risks in well-selected patients (Woods et al., 2002; Voon et al., 2006). However, DBS is not a panacea for PD and does not directly address all symptoms, particularly non-motor symptoms (Rezai et al., 2008).

## Potential Psychological Effects of DBS

The past 15 years have seen an increasing amount of research devoted to whether DBS causes psychological changes in PD patients. Such effects are plausible for at least three reasons. First, DBS may impact cognitive, emotional, behavioral, and neuropsychiatic networks because these functional networks are adjacent to the motor fronto-subcortical networks targeted by DBS (Alexander et al., 1986; Cummings, 1993; Lanciego et al., 2012; Mosley et al., 2020). Second, living with technological equipment in the brain that alters functioning may present profound challenges to identity (e.g., Hildt, 2006; Schechtman, 2010; Witt et al., 2013). Third, the potential lifestyle changes (e.g., occupational, relational) that occur following DBS may relate to shifting patterns of psychosocial functioning (Deuschl et al., 2006a).

Excellent review articles touch upon the psychological effects of DBS, focusing on issues such as impulsivity (Jahanshahi et al., 2015), psychological variables relevant to neuroethics (Gilbert et al., 2018), acute and enduring psychiatric and neuropsychiatric changes (Castrioto et al., 2014; Kurtis et al., 2017), and measurement of these and other complex changes (Ineichen et al., 2016). Some of these articles reviewed individual studies that assessed variables relevant to the field of personality psychology, such as personality traits (Houeto et al., 2002), and personality disorders (Castelli et al., 2006), however, none of the reviews focused primarily on personality changes. Thus, our central aim is to provide a brief but relatively comprehensive evaluation of this research. Before doing so, it is important to frame our review within the context of three relevant issues: (a) defining personality and (b) offering a putative neurobiological basis for personality change following DBS.

## A Multilayer Approach to Defining Personality

Personality is broadly defined as a person's characteristic and relatively stable patterns of affect, behavior, cognition, and desire (the ABCDs of personality) over time and situation (Revelle et al., 2011). There have been several efforts to organize the abundant number of diverse individual differences subsumed by the field of personality psychology (e.g., McAdams and Pals, 2006; McCrae and Costa, 2008; DeYoung, 2015; Mayer, 2015). Though the particulars of these approaches are beyond the scope of this paper, we believe that a brief overview of an influential, multilayer approach (McAdams and Pals, 2006) will help with framing and interpreting research on the effects of DBS on personality. In this approach, at the most basic layer are a relatively small number of dispositional traits such as the Big Five [extraversion, agreeableness, conscientiousness, emotional stability (or neuroticism), and openness] and their lower order facets: Traits and facets are used to describe and explain the coherence and stability of ABCD patterns across time and situation. The second layer, characteristic adaptations, comprise motivational constructs (e.g., goals, values), developmental variables (e.g., attachment styles), and social attitudes (e.g., moral attitudes, prejudice) in which ABCD patterns are contextualized by time, situation, and/or social roles. The third layer, narrative identity, includes a person's self-authored life-stories connecting ABCD patterns from the past, present, and imagined future into an integrated whole that may provide a person with a sense of unity, coherence, and purpose^1^.

The three layers theoretically apply to both normal and pathological personality characteristics (McAdams, 2020). However, whereas assessments of normal personality span the three levels, assessments of pathological personality may not. Measures of personality pathology focus predominantly on personality disorders, which are defined as “an enduring pattern of inner experience and behavior that deviates markedly from the expectations of the individual's culture, is pervasive and inflexible, has an onset in adolescence or early adulthood, is stable over time, and leads to distress or impairment” (American Psychiatric Association, p. 645). Even though personality disorders affect the whole person, and therefore may reflect dysfunction at the three layers of personality described above, assessment of personality disorders typically focuses on routine patterns of ABCDs (i.e., dispositional traits; Nuzum et al., 2019), and conceptualizations of personality pathology as maladaptive personality traits are gaining influence (e.g., Pocnet et al., 2018; Bagby and Widiger, 2020). Thus, existing personality pathology variables may be viewed as reflecting dispositional traits.

We will classify variables according to the levels of dispositional traits, characteristic adaptations, and narrative identity, when possible. We find this potentially useful for at least three reasons. First, it will illustrate which kinds of variables have received research attention and which have not, thereby possibly identifying areas in need of future research. Second, it may reveal which kinds of variables are more likely to change (for better or worse) or stay the same following DBS. Third, it may help improve the informed consent process for DBS; knowledge about what kind of personality characteristics might change could be helpful to the patient and the clinical team during discussions about the potential treatment risks and benefits.

Finally, we note two important exclusions from the multilayer framework for personality. First, this approach excludes neuropsychiatric disorders outside of personality disorders (e.g., depressive disorders, impulse control disorders, psychotic disorders, substance-related disorders, etc.). Though many psychiatric conditions entail significant and enduring disruptions in ABCDs and cannot be cleanly demarcated from personality, a thorough consideration of which disorders may include personality components is beyond the scope of this paper, and thus we chose to focus on personality disorders (and maladaptive personality traits). Second, the multilayer approach does not encompass cognitive dysfunction in areas such as attention, learning, memory, and executive function (Sahakian et al., 2015) which are known to be related to neural networks associated with variables related to personality such as initiative/drive, mood, and emotion regulation. Castrioto et al. (2014) and Kurtis et al. (2017) cover the effects of DBS on psychiatric and cognitive conditions comprehensively.

## Putative Neurological Basis of Personality Change Following DBS in PD

Without underestimating the psychosocial effects of DBS, neurobiological changes associated with DBS may be associated with changes involving any of the personality layers described above. Though DBS modulation specifically targets basal ganglia motor regions, the proximity of non-motor fiber networks to motor networks in the basal ganglia coupled with the intimate connections between the basal ganglia and limbic areas (Lanciego et al., 2012) makes it possible that DBS could modulate nonmotor neurobehavioral circuitry and functions as well. DBS for the treatment of motor symptoms of PD targets nodes along a motor cortical-basal ganglia-thalamo-cortical loop. The motor loop runs parallel to other cortico-basal ganglia-thalamo-cortical loops including a cognitive loop primarily associated with executive cognitive function (including emotion regulation) and two limbic loops originating in the orbitofrontal region (which is broadly associated with emotion) and anterior cingulate region (which is broadly associated with inhibition/disinhibition) [see Alexander et al. (1986) and Cummings (1993) for classic references]. Consequently, DBS stimulation in motor regions of the basal ganglia might theoretically spread to non-motor fiber pathways and result in neurobehavioral symptoms related to emotion regulation, motivation, and disinhibition. Case studies provide examples of these potential side-effect which are most often quickly addressed by altering stimulation parameters (e.g., Bejjani et al., 1999; Herzog et al., 2003; Wojtecki et al., 2007). Neuroimaging studies also support the role of the basal ganglia in emotional behavior and disorders more broadly [see Phillips et al. (2003a) and Phillips et al. (2003b)]. For example, regions in the basal ganglia are part of neural networks that have been linked to the personality trait of neuroticism (DeYoung et al., 2010), as well as psychiatric disorders relevant to neuroticism such as depression, anxiety disorders and phobias, and obsessive compulsive disorder (Kopell and Greenberg, 2008; Tremblay et al., 2015). Computational modeling has shown that the subthalamic nucleus is directly linked to impulsivity (Frank et al., 2007). DBS in the subthalamic has been associated with activation of regions of the limbic system, which is known to be central in emotional processing (Ulla et al., 2011). These observations just scratch the surface of potential effects of DBS on behaviors highly associated with the construct of personality.

## PD and Medications for PD May Affect Personality

A diagnosis of PD has been associated with a personality profile characterized by low levels of impulsivity, flexibility, and novelty-seeking, as well as high levels of industriousness, cautiousness, and harm avoidance [see Poletti and Bonuccelli (2012), Santangelo et al. (2017), and Cerasa (2018)]. Neuropsychiatric symptoms associated with the underlying neuropathological changes of PD, such as depression, anxiety, and apathy (Pandya et al., 2008), are commonly seen with estimates ranges from 30–40% for depression or anxiety to 17–70% for apathy (Chaudhuri et al., 2006; Aarsland et al., 2009). Such changes may affect personality ratings, especially those including negative emotions. Consequently, changes in personality over the course of DBS treatment may be difficult to disentangle from the underlying disease.

Pharmacological treatments for PD may also cause changes in personality and psychiatric symptoms. Levodopa and dopamine agonists are associated with impulsivity and reward-seeking behaviors (Lhommée et al., 2017), as well as impulse control disorder symptoms, including increased gorging of sweets, gambling/increased spending, increased hobbyism, and hypersexuality in some patients (Weintraub et al., 2010). In addition, dopaminergic medications can improve symptoms of apathy. Reductions in dopaminergic medications following successful DBS therapy have been associated with increased symptoms of apathy (Rossi et al., 2018). Relatedly, anti-depressants and anti-anxiety medications are frequently prescribed to alleviate psychological symptoms associated with PD. Changes in psychotropic medications and/or ongoing psychotropic medication management in patients may make it difficult to pinpoint the unique effects of DBS on psychiatric symptoms.

Disentangling the potential impact of underlying disease and potential medication effects on personality is further complicated by including the variable of time. As noted, PD is a progressive neurodegenerative disorder associated with the development and/or worsening of symptoms over time which may result in ongoing changes in medications. As motor symptoms increase, patients' relationships with family members and their ability to engage in important activities (e.g., work, drive) can be impacted which may contribute to personality changes (e.g., decreased feelings of confidence, sadness associated with various losses). Thus, assessment of personality changes in patients who are undergoing DBS therapy is complicated and requires a clear understanding of the underlying disease, typical neurobehavioral symptom onset timeline, and role of different treatments on relevant variables associated with the broad construct of personality.

## Review of Empirical Research

Against this complicated backdrop, we examine empirical evidence pertaining to personality change following DBS. We evaluate the types of methods used to study this question, and we summarize and integrate research findings across personality characteristics. Finally, we list a number of strategies for advancing research.

### Inclusion Criteria and Search Strategy

We included articles that met the following criteria: (a) meta-analyses and primary studies including PD patients who had undergone DBS that (b) assessed personality characteristics encompassed by the multilayer framework (McAdams and Pals, 2006) described previously. To compile our list of articles, we (a) relied on previous knowledge of the relevant research, (b) conducted searches on Google Scholar including (but not limited to) the terms “Deep Brain Stimulation,” “DBS,” “Parkinson's Disease,” “personality,” and “personality change” (both in isolation and combination), and (c) used connectedpapers.com to discover relevant papers that cited (or were cited by) articles we decided to include in the review.

### Methods for Assessing Personality Changes Following DBS

Supplementary Table 1 gives detailed descriptions of studies (and their relevant results) included in our review, grouped according to primary methods. Most research used quantitative methods, while qualitative methods are growing increasingly common. There is an emerging trend for studies to assess patient valued characteristics using a mixed-methods approach. Across methods, most studies relied only on self-reports, though a few assessed caregiver or clinician perspectives.

#### Quantitative Methods

Quantitative research on personality change comes from (a) meta-analyses and (b) primary studies employing retrospective, cross-sectional, prospective, and randomized clinical trial (RCT) designs. This research has some general limitations. Meta-analyses focused mainly on clinical measures of psychiatric symptoms rather than scales assessing normal range personality. Most retrospective, cross-sectional, and prospective studies included small sample sizes, limiting power to detect small- to moderate effect sizes. And there has been one RCT to date, which decreases the confidence with which causal claims can be made. Additionally, quantitative studies focused almost exclusively on dispositional traits (using a wide variety of measures) rather than characteristic adaptations and narrative identity.

Though quantitative research using standardized scales has generated the most knowledge about personality change following DBS, there are at least two limitations to this method that may be particularly relevant to DBS patients. First, there is no opportunity for patients to elaborate on the personal relevance of the characteristics that are assessed. Relatedly, standardized scales, because they assess predetermined constructs rather than patient-defined variables, may not capture some of the personality characteristics that are viewed as most salient to the lived experience of patients (Nisenzon et al., 2011; Kubu and Ford, 2012). Thus, standardized scales might not fully assess the rich, idiosyncratic, and nuanced experience of patients.

#### Qualitative Methods

Given the relatively rare and unique experience of undergoing DBS for PD, it is important to gain a more personalized account of perceptions of personality change. Qualitative assessments have the potential to redress the limitations noted above. Asking open-ended questions gives patients opportunities to identify important characteristics that standardized assessments exclude and provides time for elaborating regarding why those characteristics are relevant to the patient's life. The five qualitative studies in Supplementary Table 1 employed semistructured interviews and coding techniques to identify salient themes relevant to personality change: Together, these studies revealed perceived personality changes that could be categorized across dispositional traits, characteristic adaptations, and narrative identity.

The strengths noted above should be balanced against the limitations inherent in qualitative methods, such as the biases of the research team, as well as limited replicability and generalizability (Creswell and Poth, 2016). Researcher bias is inherent to qualitative methods; for instance, researchers may prefer a certain theoretical framework and tend to code for characteristics consistent with that framework. Limited replicability is due in part to intensive coding that makes only small sample sizes feasible. In turn, small sample sizes limit the types of claims that can be made; for instance, most qualitative studies in Supplementary Table 1 showed that patients perceived personality changes in positive and negative directions but did not generate claims regarding whether positive or negative changes were more likely. The limited generalizability is because open-ended responses lack the standardization of scales that allows for making valid comparisons of results across multiple samples.

#### Patient Valued Characteristics: A Mixed Methods Approach

An emerging trend in DBS research, the use of quantitative measures of patient-valued characteristics [see, e.g., Kubu et al. (2019)], attempts to blend the strengths and mitigate the limitations of quantitative and qualitative research. Patients (and sometimes their caregivers) are consulted about what constitutes important values, goals, and/or traits, which may be rated on quantitative scales over the course of treatment (Kubu and Ford, 2012). Thus, this method may have the potential to capture what patients care about qualitatively and produce replicable and generalizable quantitative data. Other areas of research are beginning to incorporate patients' lived experiences into protocols to determine what patients value (Ghosh et al., 2021). Few studies have used mixed methods to study patient valued characteristics, and all focused on characteristic adaptations.

### Summary of Findings

Research has assessed a wide range of personality variables (see Table 1). Only a few studies at most address any one variable, and thus all claims below may be viewed as preliminary. Most research involved relatively small sample sizes, and thus only the strongest effects are likely to have been detected. Furthermore, the lack of RCTs hampers causal inferences; though the focal outcome variables of RCTs will likely always be more directly clinically relevant (e.g., neuropsychiatric symptoms) than personality changes, future research may assess personality in conjunction with focal outcomes. With these caveats in mind, we turn to a review of extant findings.

**Table 1:** Summary of findings organized by layers of personality and variables.

**Layer of personality**	**Variables**	**Associations between DBS and variables**
Dispositional traits		
	Cloninger's personality theory traits	
	Novelty-seeking	–/0: Lhommée et al. (2017)—Quant/P 0: Houeto et al. (2006)—Quant/P; Pham et al. (2015)—Quant/P 0/+: Fassino et al. (2010)—Quant/P
	Harm avoidance	0: Houeto et al. (2006)—Quant/P; Fassino et al. (2010)—Quant/P; Pham et al. (2015)—Quant/P +: Lewis et al. (2015)—Quant/P
	Reward dependance	–/0: Lewis et al. (2015)—Quant/P 0: Houeto et al. (2006)—Quant/P; Fassino et al. (2010)—Quant/P; Pham et al. (2015)—Quant/P
	Persistence	–: Pham et al. (2015)—Quant/P 0: Houeto et al. (2006)—Quant/P; Fassino et al. (2010)—Quant/P
	Self-directedness	0: Houeto et al. (2006)—Quant/P; Fassino et al. (2010)—Quant/P; Pham et al. (2015)—Quant/P
	Cooperativeness	0: Houeto et al. (2006)—Quant/P; Fassino et al. (2010)—Quant/P; Pham et al. (2015)—Quant/P
	Self-transcendence	–: Pham et al. (2015)—Quant/P 0: Houeto et al. (2006)—Quant/P 0/+: Fassino et al. (2010)—Quant/P
	Big five traits	
	Extraversion	–/0: Boel et al. (2016)—Quant/RCT
	Agreeableness	–/0: Boel et al. (2016)—Quant/RCT
	Conscientiousness	0: Boel et al. (2016)—Quant/RCT
	Neuroticism	0: Pham et al. (2015)—Quant/P; Boel et al. (2016)—Quant/RCT
	Openness	–/0: Boel et al. (2016)—Quant/RCT
	Affective/behavioral traits	
	Negative affective traits	–/+: Gilbert (2018)—Qual; Thomson et al. (2019)—Qual 0/+: Thomson et al. (2019)—Qual
	Positive affective traits	–/+: Gilbert (2018)—Qual; Thomson et al. (2020)—Qual
	Aggressiveness	0: Temel et al. (2006)—Quant/MA
	Anxiety	0: Castelli et al. (2006)—Quant/P
	Apathy	0: Temel et al. (2006)—Quant/MA; Appleby et al. (2007)—Quant/MA +: Denheyer et al. (2009)—Quant/Ret; Gilbert (2012)—Qual
	Hypersexuality	0: Temel et al. (2006)—Quant/MA; Appleby et al. (2007)—Quant/MA
	Hypomania	+: Lewis et al. (2015)—Quant/P
	Impulsivity	0: Lewis et al. (2015)—Quant/P 0/+: Pham et al. (2015)—Quant/P +: Hälbig et al. (2009)—Quant/CS
	Personality pathology	
	General personality pathology	0: Temel et al. (2006)—Quant/MA; Appleby et al. (2007)—Quant/MA; –/+ : Houeto et al. (2002)—Quant/Ret
	Personality disorders: obsessive-compulsive, paranoid	–: Castelli et al. (2006)—Quant/P
	Personality disorders: avoidant, dependent, passive-aggressive, self-frustrating, schizotypal, schizoid, histrionic, narcissistic, borderline, antisocial	0: Castelli et al. (2006)—Quant/P
Characteristic adaptations	Ability to pursue leisure activities	+: Törnqvist et al. (2007)—PVC/P; Kubu et al. (2017a,b)—PVC/P
	Ability to pursue social activities	+: Törnqvist et al. (2007)—PVC/P; Kubu et al. (2017a,b)—PVC/P
	Ability to pursue work activities	+: Törnqvist et al. (2007)—PVC/P; Kubu et al. (2017a,b)—PVC/P
	Difficulties with relationships	–/+: Gilbert (2018)—Qual +: Gilbert (2012)—Qual
	Difficulties with routine behaviors	–/+: Gilbert (2018)—Qual +: Gilbert (2012)—Qual
	Leisure/social/work goal importance	+/–: Kubu et al. (2018)—PVC/P
	Positive relationship-specific coping strategies	+: Baumann-Vogel et al. (2020)—Quant/CS
Narrative identity	Difficulties with self-image/self-estrangement	–/+: Gilbert et al. (2017)—Qual; Gilbert (2018)—Qual +: Gilbert (2012)—Qual

“–”evidence for negative association; “0”: no evidence for directional association; “+”: evidence for positive association; when –, 0, or + are separated by “/”, the study provided mixed evidence for associations [e.g., Lewis et al. (2015) provided evidence for negative and null associations between DBS and novelty-seeking]; References are followed by “–Method/Design”; “Quant” = quantitative methods; “Qual” = qualitative methods; “PVC” = patient valued characteristics (mixed methods); “MA” = meta-analysis; “Ret” = retrospective design; “CS” = Cross-sectional design; “P” = prospective design; “RCT” = randomized clinical trial.

By far the most work has been done at the level of dispositional traits. Given the popularity of assessing the Big Five in general (John et al., 2008), it is somewhat surprising that comparatively more research used inventories relevant to Cloninger's personality theory (Cloninger et al., 1993), such as the Temperament Character Inventory (Cloninger et al., 1994) and Tridimensional Personality Questionnaire (Cloninger et al., 1991). Outside of those approaches, several studies examined other affective (e.g., positive and negative affect, anxiety, and apathy) and behavioral (e.g., aggressiveness, hypersexuality, and impulsiveness) traits, and meta-analyses and a couple of other studies assessed personality pathology. Perhaps the most consistent finding is that, across normal range traits (Cloninger's traits, Big Five traits, and other affective/behavioral traits) and personality pathology, many studies found no evidence of change or mixed evidence regarding change, with an occasional study reporting only an increase or decrease in a given trait. Thus, there is very little evidence that any dispositional traits change following DBS.

Though research on characteristic adaptations is relatively sparse, this work shows somewhat consistent associations between DBS and improvements in abilities to carry out a variety of role-specific behaviors across a number of domains: routine, work, social/relational, and leisure. These findings point to the possibility that perhaps personality changes following DBS may be more common at level of characteristic adaptations than dispositional traits. We are hesitant to make stronger claims until more research has been done on characteristic adaptations.

Very little work has tapped constructs relevant to narrative identity. Findings from two qualitative studies suggest that issues around self-image and self-estrangement may be common for DBS patients: Some patients perceived improvements in these areas, whereas others perceived deficits.

## Ways Forward

In addition to calling for larger sample sizes and RCTs that assess personality variables (while recognizing the challenges of instituting those design features for the population in question), we offer six suggestions for advancing research. First, we suggest taking a step back from studies that typically rely on one personality inventory (or one qualitative method) to assess personality change; rather, it may be fruitful to frame research within integrative personality frameworks that define and describe interrelations between different parts of personality, such as traits, contextualized roles, and identity (e.g., McAdams and Pals, 2006; McCrae and Costa, 2008; Mayer, 2015). Doing so may help to organize current findings and identify aspects of personality that have previously been overlooked. Second, it is clear that dispositional traits assessed by quantitative instruments have received the most research attention, so we encourage future studies on more contextualized aspects of personality and identity while taking into account the patient's opinions on valued personality characteristics. Third, most research has relied on the patient's perceptions of personality alone or, in the case of personality pathology, a clinician's rating; because the patient, caregivers, and clinicians all provide invaluable viewpoints, we encourage studies assessing personality from multiple perspectives (Goering et al., 2017). Fourth, as DBS for PD spans multiple areas of expertise in scholarship and practice (e.g., medicine, surgery, neuroscience, clinical neuropsychology, psychopathology, personality, and neuroethics just to name a few), interdisciplinary scholarship is a necessity (Kubu and Ford, 2012; Kubu et al., 2019). Fifth, and related to the prior four points, we believe that perhaps the most crucial advances will come from interdisciplinary efforts to design and validate new assessments capable of measuring complex personality changes across various constructs and multiple raters (Kubu and Ford, 2012; Ineichen et al., 2016). Sixth, integration of research on DBS and personality change for other conditions, such as epilepsy (Gilbert, 2012), primary dystonia (Hariz et al., 2011), Tourette's Syndrome (Schoenberg et al., 2015), OCD (de Haan et al., 2013, 2015), and others will help to identify DBS effects that generalize and those that are unique to PD.

In conclusion we offer two ways in which the findings of this review may be applied. First, we advocate for interdisciplinary work applying findings regarding personality change to improve the informed consent process; patient expectations and goals regarding personality change can be assessed and compared to the current state of knowledge, which may lead to enhanced communication and collaboration among patients and interdisciplinary treatment teams and ultimately better decisions about whether to pursue DBS. Further, the findings may help treatment teams to assess and manage patient expectations throughout the course of treatment; guiding patients to endorse and then maintain realistic expectations regarding personality change may guard against overly negative and potentially harmful reactions to unmet expectations.

## Author Contributions

JW wrote the manuscript. CK and AM revised the manuscript and provided critical comments on the manuscript. JZ and MM provided comments on the manuscript. All authors contributed to the article and approved the submitted version.

## Conflict of Interest

The authors declare that the research was conducted in the absence of any commercial or financial relationships that could be construed as a potential conflict of interest.

## Publisher's Note

All claims expressed in this article are solely those of the authors and do not necessarily represent those of their affiliated organizations, or those of the publisher, the editors and the reviewers. Any product that may be evaluated in this article, or claim that may be made by its manufacturer, is not guaranteed or endorsed by the publisher.

## Footnotes

^1^We are intentionally excluding the other layers in this framework (evolution, culture) because they do not focus on individual differences.

## References

[B1] Aarsland D. Marsh L. Schrag A. (2009). Neuropsychiatric symptoms in Parkinson's disease. Mov. Disord. Off. J. Mov. Disord. Soc. 24, 2175–2186. 10.1002/mds.22589 PMC2787875 19768724

[B2] Alexander G. E. De Long M. R. Strick P. L. (1986). Parallel organization of functionally segregated circuits linking basal ganglia and cortex. Ann. Rev. Neurosci. 9, 357–381. 10.1146/annurev.ne.09.030186.002041 3085570

[B3] Appleby B. S. Duggan P. S. Regenberg A. Rabins P. V. (2007). Psychiatric and neuropsychiatric adverse events associated with deep brain stimulation: a meta-analysis of ten years' experience. Mov. Disord. Off. J. Mov. Disord. Soc. 22, 1722–1728. 10.1002/mds.21551 17721929

[B4] Bagby R. M. Widiger T. A. (2020). Assessment of the ICD-11 dimensional trait model: an introduction to the special section. Psychol. Assess. 32, 1–7. 10.1037/pas0000785 31750681

[B5] Barrash J. Anderson S. W. Jones R. D. Tranel D. (1997). The Iowa rating scales of personality change: reliability and validity. J. Int. Neuropsychol. Soc. 3, 27–28.

[B7] Baumann-Vogel H. Bodenmann G. Schmid J. Waldvogel D. Ineichen C. Baumann C. R. (2020). Partners' view after subthalamic deep brain stimulation: better relationships despite patients being less active. Clin. Parkinson. Relat. Disord. 3:100052. 10.1016/j.prdoa.2020.100052 PMC8298790 34316635

[B8] Bejjani B. P. Damier P. Arnulf I. Thivard L. Bonnet A. M. Dormont D.. (1999). Transient acute depression induced by high-frequency deep-brain stimulation. N. Engl. J. Med. 340, 1476–1480. 10.1056/NEJM199905133401905 10320386

[B9] Bodenmann G. (2008). Dyadisches Coping Inventar (DCI) [Dyadic Coping Inventory]. Bern: Huber.

[B10] Boel J. A. Odekerken V. J. Geurtsen G. J. Schmand B. A. Cath D. C. Figee M.. (2016). Psychiatric and social outcome after deep brain stimulation for advanced Parkinson's disease. Mov. Disord. 31, 409–413. 10.1002/mds.26468 26660279

[B15] Braak H. Bohl J. R. Muller C. M. Rub U. de Vos R. A. I. Del Tredici K. (2006). Stanley Fahn lecture 2005: the staging procedure for the inclusion body pathology associated with sporadic Parkinson's disease reconsidered. Mov. Disord. 21, 2042–2050. 10.1002/mds.21065 17078043

[B17] Braun V. Clarke V. (2006). Using thematic analysis in psychology. Qual. Res. Psychol, 3, 77–101. 10.1191/1478088706qp063oa

[B19] Castelli L. Perozzo P. Zibetti M. Crivelli B. Morabito U. Lanotte M.. (2006). Chronic deep brain stimulation of the subthalamic nucleus for Parkinson's disease: effects on cognition, mood, anxiety and personality traits. Eur. Neurol. 55, 136–144. 10.1159/000093213 16682797

[B20] Castrioto A. Lhommée E. Moro E. Krack P. (2014). Mood and behavioural effects of subthalamic stimulation in Parkinson's disease. Lancet Neurol. 13, 287–305. 10.1016/S1474-4422(13)70294-1 24556007

[B21] Cerasa A. (2018). Re-examining the Parkinsonian personality hypothesis: a systematic review. Pers. Individ. Dif. 130, 41–50. 10.1016/j.paid.2018.03.045

[B22] Chaudhuri K. R. Healy D. G. Schapira A. H. (2006). Non-motor symptoms of Parkinson's disease: diagnosis and management. Lancet Neurol. 5, 235–245. 10.1016/S1474-4422(06)70373-8 16488379

[B24] Cloninger C. Svrakic D. M. Przybeck T. R. (1993). A psychobiological model of temperament and character. Arch. Gen. Psychiatry 50, 975–990. 8250684 10.1001/archpsyc.1993.01820240059008

[B25] Cloninger C. R. (1999). The Temperament and Character Inventory—Revised. St Louis, MO: Center for Psychobiology of Personality, Washington University.

[B26] Cloninger C. R. Przybeck T. R. Svrakic D. M. (1991). The tridimensional personality questionnaire: US normative data. Psychol. Rep. 69, 1047–1057. 1784653 10.2466/pr0.1991.69.3.1047

[B27] Cloninger C. R. Przybeck T. R. Svrakic D. M. Wetzel R. D. (1994). The Temperament and Character Inventory (TCI): A Guide to Its Development and Use. St Louis, MO: Center for Psychobiology of Personality, Washington University.

[B28] Creswell J. W. Poth C. N. (2016). Qualitative Inquiry and Research Design: Choosing Among Five Approaches. Thousand Oaks, CA: Sage publications.

[B29] Cummings J. L. (1993). Frontal-subcortical circuits and human behavior. Arch. Neurol. 50, 873–880. 10.1001/archneur.1993.00540080076020 8352676

[B31] de Haan S. Rietveld E. Stokhof M. Denys D. (2013). The phenomenology of deep brain stimulation-induced changes in OCD: an enactive affordance-based model. Front. Hum. Neurosci. 7:653. 10.3389/fnhum.2013.00653 24133438 PMC3794192

[B32] de Haan S. Rietveld E. Stokhof M. Denys D. (2015). Effects of deep brain stimulation on the lived experience of Obsessive-Compulsive Disorder patients: in-depth interviews with 18 patients. PLoS ONE 10:e0135524. 10.1371/journal.pone.0135524 26312488 PMC4552296

[B33] Denheyer M. Kiss Z. H. Haffenden A. M. (2009). Behavioral effects of subthalamic deep brain stimulation in Parkinson's disease. Neuropsychologia 47, 3203–3209. 10.1016/j.neuropsychologia.2009.07.022 19664645

[B34] Deuschl G. Herzog J. Kleiner-Fisman G. Kubu C. Lozano A. M. Lyons K. E.. (2006a). Deep brain stimulation: postoperative issues. Mov. Disord. Off. J. Mov. Disord. Soc. 21, S219–S237. 10.1002/mds.20957 16810719

[B35] Deuschl G. Schade-Brittinger C. Krack P. Volkmann J Schäfer H, Bötzel K. (2006b). A randomized trial of deep-brain stimulation for Parkinson's disease. N. Engl. J. Med. 355, 896–908. 10.1056/NEJMoa060281 16943402

[B36] DeYoung C. G. (2015). Cybernetic big five theory. J. Res. Pers. 56, 33–58. 10.1016/j.jrp.2014.07.004

[B37] DeYoung C. G. Hirsh J. B. Shane M. S. Papademetris X. Rajeevan N. Gray J. R. (2010). Testing predictions from personality neuroscience. Psychol. Sci. 21:820. 10.1177/0956797610370159 20435951 PMC3049165

[B38] Dickson D. W. (2012). Parkinson's disease and parkinsonism: neuropathology. Cold Spring Harb. Perspect. Med. 2:a009258. 10.1101/cshperspect.a009258 22908195 PMC3405828

[B41] Fassino S. Daga G. A. Gramaglia C. Piero A. Zibetti M. Castelli L.. (2010). Novelty-Seeking in Parkinson's Disease after deep brain stimulation of the subthalamic nucleus: a case-control study. Psychosomatics 51, 62–67. 10.1016/S0033-3182(10)70660-5 20118442

[B43] Frank M. J. Samanta J. Moustafa A. A. Sherman S. J. (2007). Hold your horses: impulsivity, deep brain stimulation, and medication in parkinsonism. Science 318, 1309–1312. 10.1126/science.1146157 17962524

[B45] Ghosh C. C. McVicar D. Davidson G. Shannon C. (2021). Measuring diagnostic heterogeneity using text-mining of the lived experiences of patients. BMC Psychiatry 21, 1–12. 10.1186/s12888-021-03044-1 33509154 PMC7842026

[B46] Gilbert F. (2012). The burden of normality: from “chronically ill” to “symptom free.” New ethical challenges for deep brain stimulation postoperative treatment. J. Med. Ethics 38, 408–412. 10.1136/medethics-2011-100044 22431560

[B47] Gilbert F. (2018). Deep brain stimulation: inducing self-estrangement. Neuroethics 11, 157–165. 10.1007/s12152-017-9334-7

[B48] Gilbert F. Goddard E. Viaña J. N. M. Carter A. Horne M. (2017). I miss being me: phenomenological effects of deep brain stimulation. AJOB Neurosci. 8, 96–109. 10.1080/21507740.2017.1320319

[B49] Gilbert F. Viaña J. N. Ineichen C. (2020). Deflating the deep brain stimulation causes personality changes bubble: the authors reply. Neuroethics. 10.1007/s12152-020-09437-5

[B50] Gilbert F. Viaña J. N. M. Ineichen C. (2018). Deflating the “DBS causes personality changes” bubble. Neuroethics. 10.1007/s12152-018-9386-3

[B51] Goering S. Brown T. Alsarraf J. (2017). Others' contributions to narrative identity matter. AJOB Neurosci. 8, 176–178. 10.1080/21507740.2017.1366586

[B53] Groiss S. J. Wojtecki L. Südmeyer M. Schnitzler A. (2009). Deep brain stimulation in Parkinson's disease. Ther. Adv. Neurol. Disord. 2, 20–28. 10.1177/1756285609339382 21180627 PMC3002606

[B54] Hälbig T. D. Tse W. Frisina P. G. Baker B. R. Hollander E. Shapiro H.. (2009). Subthalamic deep brain stimulation and impulse control in Parkinson's disease. Eur. J. Neurol. 16, 493–497. 10.1111/j.1468-1331.2008.02509.x 19236471

[B57] Hariz G.-M. Limousin P. Tisch S. Jahanshahi M. Fjellman-Wiklund A. (2011). Patients' perceptions of life shift after deep brain stimulation for primary dystonia-a qualitative study. Mov. Disord. 26, 2101–2106. 10.1002/mds.23796 21626564

[B58] Herzog J. Reiff J. Krack P. Witt K. Schrader B. Müller D.. (2003). Manic episode with psychotic symptoms induced by subthalamic nucleus stimulation in a patient with Parkinson's disease. Mov. Disord. Off. J. Mov. Disord. Soc. 18, 1382–1384. 10.1002/mds.10530 14639687

[B59] Hildt E. (2006). Electrodes in the brain: some anthropological and ethical aspects of deep brain stimulation. Int. Rev. Inform. Ethics 5, 33–38. Available online at: http://informationethics.ca/index.php/irie/article/view/193

[B61] Houeto J.-L. Mallet L. Mesnage V. Tezenas du Montcel S. Béhar C. Gargiulo M.. (2006). Subthalamic stimulation in Parkinson Disease: behavior and social adaptation. JAMA Neurol. 63, 1090–1095. 10.1001/archneur.63.8.1090 16908734

[B62] Houeto J. L. Mesnage V. Mallet L. Pillon B. Gargiulo M. du Moncel S. T.. (2002). Behavioural disorders, Parkinson's disease and subthalamic stimulation. J. Neurol. Neurosurg. Psychiatry 72:701. 10.1136/jnnp.72.6.701 12023409 PMC1737905

[B63] Ineichen C. Baumann-Vogel H. Christen M. (2016). Deep brain stimulation: in search of reliable instruments for assessing complex personality-related changes. Brain Sci. 6:40. 10.3390/brainsci6030040 27618110 PMC5039469

[B64] Jahanshahi M. Obeso I. Baunez C. Alegre M. Krack P. (2015). Parkinson's Disease, the subthalamic nucleus, inhibition, and impulsivity. Mov. Disord. 30, 128–140. 10.1002/mds.26049 25297382

[B65] Jankovic J. (2008). Parkinson's disease: clinical features and diagnosis. J. Neurol. Neurosurg. Psychiatry 79, 368–376. 10.1136/jnnp.2007.131045 18344392

[B66] John O. P. Robins R. W. Pervin L. A. (2008). Handbook of Personality: Theory and Research, 3rd edn. New York, NY: Guilford Press.

[B67] Kliem S. Job A. K. Kröger C. Bodenmann G. Stöbel-Richter Y. Hahlweg K.. (2012). Entwicklung und Normierung einer Kurzform des Partnerschaftsfragebogens (PFB-K) an einer repräsentativen deutschen Stichprobe. Zeitschrift für Klinische Psychologie und Psychotherapie. 41, 81−89. 10.1026/1616-3443/a000135

[B68] Kopell B. H. Greenberg B. D. (2008). Anatomy and physiology of the basal ganglia: implications for DBS in psychiatry. Neurosci. Biobehav. Rev. 32, 408–422. 10.1016/j.neubiorev.2007.07.004 17854894

[B69] Kubu C. S. Cooper S. E. Machado A. Frazier T. Vitek J. Ford P. J. (2017a). Insights gleaned by measuring patients' stated goals for DBS: more than tremor. Neurology 88, 124–130. 10.1212/WNL.0000000000003485 27913696 PMC5224715

[B70] Kubu C. S. Ford P. (2012). Beyond mere symptom relief in deep brain stimulation: an ethical obligation for multifaceted assessment of outcome. AJOB Neurosci. 3, 44–49. 10.1080/21507740.2011.633960 22737593 PMC3377486

[B71] Kubu C. S. Ford P. J. Lapin B. (2017b). Patients' Perceptions of Personality Change in Parkinson's Disease and Following Deep Brain Stimulation. Washington, DC: International Neuroethics Society meeting.

[B72] Kubu C. S. Ford P. J. Wilt J. A. Merner A. R. Montpetite M. Zeigler J.. (2019). Pragmatism and the importance of interdisciplinary teams in investigating personality changes following DBS. Neuroethics 2019:10.1007/s12152-019-09418-3. 10.1007/s12152-019-09418-3 32952741 PMC7500511

[B73] Kubu C. S. Frazier T. Cooper S. E. Machado A. Vitek J. Ford P. J. (2018). Patients' shifting goals for deep brain stimulation and informed consent. Neurology 91, e472–e478. 10.1212/WNL.0000000000005917 29959262 PMC6093764

[B74] Kurtis M. M. Rajah T. Delgado L. F. Dafsari H. S. (2017). The effect of deep brain stimulation on the non-motor symptoms of Parkinson's disease: a critical review of the current evidence. NPJ Parkinson's Dis. 3, 1–12. 10.1038/npjparkd.2016.24 28725706 PMC5516616

[B75] Lanciego J. L. Luquin N. Obeso J. A. (2012). Functional neuroanatomy of the basal ganglia. Cold Spring Harb. Perspectiv. Med. 2:a009621. 10.1101/cshperspect.a009621 PMC3543080 23071379

[B77] Lewis C. J. Maier F. Horstkötter N. Zywczok A. Witt K. Eggers C.. (2015). Subjectively perceived personality and mood changes associated with subthalamic stimulation in patients with Parkinson's disease. Psychol. Med. 45, 73–85. 10.1017/S0033291714001081 25066623

[B78] Lhommée E. Boyer F. Wack M. Pélissier P. Klinger H. Schmitt E.. (2017). Personality, dopamine, and Parkinson's disease: insights from subthalamic stimulation. Mov. Disord. 32, 1191–1200. 10.1002/mds.27065 28643887

[B79] Limousin P. Foltynie T. (2019). Long-term outcomes of deep brain stimulation in Parkinson disease. Nat. Rev. Neurol. 15, 234–242. 10.1038/s41582-019-0145-9 30778210

[B80] Löhle M. Storch A. Reichmann H. (2009). Beyond tremor and rigidity: non-motor features of Parkinson's disease. J. Neural Transmission 116:1483. 10.1007/s00702-009-0274-1 19680598

[B83] Mayer J. D. (2015). The personality systems framework: current theory and development. J. Res. Personal. 56, 4–14. 10.1016/j.jrp.2015.01.001

[B84] McAdams D. P. (2020). Psychopathology and the self: human actors, agents, and authors. J. Personal. 88, 146–155. 10.1111/jopy.12496 31206660

[B85] McAdams D. P. Pals J. L. (2006). A new big five: fundamental principles for an integrative science of personality. Am. Psychol. 61, 204–217. 10.1037/0003-066X.61.3.204 16594837

[B86] McCrae R. R. Costa P. T. (2008). “The five-factor theory of personality,” in Handbook of Personality: Theory and Research, 3 edn, eds O. P. John, R. W. Robins, and L. A. Pervin (Guilford Press), 159–181.

[B90] Mosley P. E. Paliwal S. Robinson K. Coyne T. Silburn P. Tittgemeyer M.. (2020). The structural connectivity of subthalamic deep brain stimulation correlates with impulsivity in Parkinson's disease. Brain 143, 2235–2254. 10.1093/brain/awaa148 32568370

[B91] Nisenzon A. N. Robinson M. E. Bowers D. Banou E. Malaty I. Okun M. S. (2011). Measurement of patient-centered outcomes in Parkinson's disease: what do patients really want from their treatment? Parkinson. Relat. Disord. 17, 89–94. 10.1016/j.parkreldis.2010.09.005 20952243 PMC3034811

[B92] Nuzum H. Shapiro J. L. Clark L. A. (2019). Affect, behavior, and cognition in personality and functioning: an item-content approach to clarifying empirical overlap. Psychol. Assess. 31, 905–912. 10.1037/pas0000712 30869967

[B93] Pandya M. Kubu C. Giroux M. (2008). The many faces of Parkinson's disease: not just a movement disorder. Cleveland Clin. J. Med. 75, 856–864. 10.3949/ccjm.75a.07005 19088004

[B94] Patton J. H. Stanford M. S. Barratt E. S. (1995). Factor structure of the Barratt impulsiveness scale. J. Clin. Psychol. 51, 768–774. 10.1002/1097-4679(199511)51:6<768::AID-JCLP2270510607>3.0.CO;2-1 8778124

[B95] Pham U. Solbakk A.-K. Skogseid I.-M. Toft M. Pripp A. H. Konglund A. E.. (2015). Personality changes after deep brain stimulation in Parkinson's Disease. Parkinson's Dis. 7:490507. 10.1155/2015/490507 25705545 PMC4325225

[B96] Phillips M. L. Drevets W. C. Rauch S. L. Lane R. (2003a). Neurobiology of emotion perception I: the neural basis of normal emotion perception. Biol. Psychiatry 54, 504–514. 10.1016/S0006-3223(03)00168-9 12946879

[B97] Phillips M. L. Drevets W. C. Rauch S. L. Lane R. (2003b). Neurobiology of emotion perception II: implications for major psychiatric disorders. Biol. Psychiatry 54, 515–528. 10.1016/S0006-3223(03)00171-9 12946880

[B98] Pocnet C. Antonietti J.-P. Handschin P. Massoudi K. Rossier J. (2018). The many faces of personality: the DSM-5 dimensional and categorical models and the five-factor model. Personal. Individ. Diff. 121, 11–18. 10.1016/j.paid.2017.09.005

[B99] Poletti M. Bonuccelli U. (2012). Personality traits in patients with Parkinson's disease: assessment and clinical implications. J. Neurol. 259, 1029–1038. 10.1007/s00415-011-6302-8 22083431

[B100] Pringsheim T. Jette N. Frolkis A. Steeves T. D. (2014). The prevalence of Parkinson's disease: a systematic review and meta-analysis. Mov. Disord. 29, 1583–1590. 10.1002/mds.25945 24976103

[B101] Pugh J. (2019). No going back? Reversibility and why it matters for deep brain stimulation. J. Med. Ethics 45, 225–230. 10.1136/medethics-2018-105139 30630971 PMC6582822

[B103] Revelle W. Wilt J. Condon D. M. (2011). “Individual differences and differential psychology: a brief history and prospect,” in The Wiley-Blackwell Handbook of Individual Differences, 2nd edn, eds T. Chamorro-Premuzic, A. Furnham, and S. von Stumm (Wiley-Blackwell), 3–38. 10.1002/9781444343120.ch1

[B104] Rezai A. R. Machado A. G. Deogaonkar M. Azmi H. Kubu C. Boulis N. M. (2008). Surgery for movement disorders. Neurosurgery 62(Suppl.2), 809–839. 10.1227/01.neu.0000316285.52865.53 18596424

[B105] Rogers G. Davies D. Pink J. Cooper P. (2017). Parkinson's disease: summary of updated NICE guidance. BMJ 358:j1951. 10.1136/bmj.j1951 28751362

[B106] Rossi M. Bruno V. Arena J. Cammarota Á. Merello M. (2018). Challenges in PD patient management after DBS: a pragmatic review. Mov. Disord. Clin. Pract. 5, 246–254 10.1002/mdc3.12592 30363375 PMC6174419

[B107] Sahakian B. J. Bruhl A. B. Cook J. Killikelly C. Savulich G. Piercy T.. (2015). The impact of neuroscience on society: cognitive enhancement in neuropsychiatric disorders and in healthy people. Philos. Trans. Royal Soc. B Biol. Sci. 370:20140214. 10.1098/rstb.2014.0214 26240429 PMC4528826

[B109] Santangelo G. Piscopo F. Barone P. Vitale C. (2017). Personality in Parkinson's disease: clinical, behavioural and cognitive correlates. J. Neurol. Sci. 374, 17–25. 10.1016/j.jns.2017.01.013 28087060

[B110] Schechtman M. (2010). Philosophical reflections on narrative and deep brain stimulation. J. Clin. Ethics 21:133. 20866019

[B111] Schoenberg M. R. Maddux B. N. Riley D. E. Whitney C. M. Ogrocki P. K. Gould D.. (2015). Five-months-postoperative neuropsychological outcome from a pilot prospective randomized clinical trial of thalamic deep brain stimulation for Tourette Syndrome. Neuromodulation 18, 97–104. 10.1111/ner.12233 25250712

[B115] Spielberger C. D. Gorsuch R. L. Luchene R. E. (1970). The State-Trait Anxiety Inventory. Palo Alto, CA: Consulting Psychologists Press.

[B116] Spitzer R. L. Williams J. B. Gibbon M. First M. B. (1992). The structured clinical interview for DSM-III-R (SCID): I: history, rationale, and description. Archiv. Gen. Psychiatry 49, 624–629. 1637252 10.1001/archpsyc.1992.01820080032005

[B118] Temel Y. Kessels A. Tan S. Topdag A. Boon P. Visser-Vandewalle V. (2006). Behavioural changes after bilateral subthalamic stimulation in advanced Parkinson disease: a systematic review. Parkinson. Relat. Disord. 12, 265–272. 10.1016/j.parkreldis.2006.01.004 16621661

[B119] Thomson C. J. Segrave R. A. Carter A. (2019). Changes in personality associated with deep brain stimulation: a qualitative evaluation of clinician perspectives. Neuroethics 2, 1–16. 10.1007/s12152-019-09419-2

[B120] Thomson C. J. Segrave R. A. Racine E. Warren N. Thyagarajan D. Carter A. (2020). “He's back so I'm not alone”: the impact of deep brain stimulation on personality, self, and relationships in Parkinson's Disease. Qualitat. Health Res. 2020:1049732320951144. 10.1177/1049732320951144 32856559

[B121] Törnqvist A. Ahlström G. Widner H. Rehncrona S. (2007). Fulfilment of patients' goals after thalamic deep brain stimulation: a follow-up study. Parkinson. Relat. Disord. 13, 29–34. 10.1016/j.parkreldis.2006.06.005 16928465

[B122] Tremblay L. Worbe Y. Thobois S. Sgambato-Faure V. Féger J. (2015). Selective dysfunction of basal ganglia subterritories: from movement to behavioral disorders. Mov. Disord. 30, 1155–1170. 10.1002/mds.26199 25772380

[B124] Ulla M. Thobois S. Llorca P.-M. Derost P. Lemaire J.-J. Chereau-Boudet I.. (2011). Contact dependent reproducible hypomania induced by deep brain stimulation in Parkinson's disease: clinical, anatomical and functional imaging study. J. Neurol. Neurosurg. Psychiatry 82, 607–614. 10.1136/jnnp.2009.199323 21047882

[B125] Voon V. Kubu C. S. Krack P. Houeto J.-L. Troster A. I. (2006). Neuropsychiatric and cognitive issues in the evaluation of Parkinson's disease. Mov. Disord. 21, S305–S327. 10.1002/mds.20963 16810676

[B126] Weaver F. M. Follett I. Stern M.. (2009). Bilateral deep brain stimulation vs. best medical therapy for patients with advanced Parkinson disease: a randomized controlled trial. JAMA 301, 63–73. 10.1001/jama.2008.929 19126811 PMC2814800

[B127] Weintraub D. Koester J. Potenza M. N. Siderowf A. D. Stacy M. Voon V.. (2010). Impulse control disorder in Parkinson disease. A cross-sectional study of 3090 patients. Archiv. Neurol. 67, 589–595. 10.1001/archneurol.2010.65 20457959

[B128] Whiteside S. P. Lynam D. R. (2001). The Five Factor Model and impulsivity: using a structural model of personality to understand impulsivity. Personal. Individ. Diff. 30, 669–689. 10.1016/S0191-8869(00)00064-7

[B131] Witt K. Kuhn J. Timmermann L. Zurowski M. Woopen C. (2013). Deep brain stimulation and the search for identity. Neuroethics 6, 499–511. 10.1007/s12152-011-9100-1 24273620 PMC3825601

[B132] Wojtecki L. Nickel J. Timmermann L. Maarouf M. Südmeyer M. Schneider F.. (2007). Pathological crying induced by deep brain stimulation. Mov. Disord. Off. J. Mov. Disord. Soc. 22, 1314–1316. 10.1002/mds.21266 17534982

[B133] Woods S. P. Fields J. A. Troster A. I. (2002). Neuropsychological sequelae of subthalamic nucleus deep brain stimulation in Parkinson's disease: a critical review. Neuropsychol. Rev. 12, 111–126. 10.1023/A:1016806711705 12371602

