# Corrigendum: Does personality change follow deep brain stimulation in Parkinson's disease patients?

**DOI:** 10.3389/fpsyg.2023.1235029

**Published:** 2023-07-12

**Authors:** Joshua A. Wilt, Amanda R. Merner, Jaclyn Zeigler, Michelle Montpetite, Cynthia S. Kubu

**Affiliations:** ^1^Department of Psychological Sciences, Case Western Reserve University, Cleveland, OH, United States; ^2^Department of Neurology, Cleveland Clinic, Cleveland, OH, United States; ^3^Cleveland Clinic Lerner College of Medicine of Case Western Reserve University, Cleveland, OH, United States

**Keywords:** personality, deep brain stimulation, personality change, Parkinson's disease, personality pathology

In the published article, there was an error in **Table 1**, “Summary of findings organized by layers of personality and variables.” as published. The table was missing the material on Characteristic adaptations and Narrative identity. The corrected Table 1 and its caption are shown below.

**Table 1:** Summary of findings organized by layers of personality and variables.

**Layer of personality**	**Variables**	**Associations between DBS and variables**
Dispositional traits		
	Cloninger's personality theory traits	
	Novelty-seeking	–/0: Lhommée et al. (2017)—Quant/P 0: Houeto et al. (2006)—Quant/P; Pham et al. (2015)—Quant/P 0/+: Fassino et al. (2010)—Quant/P
	Harm avoidance	0: Houeto et al. (2006)—Quant/P; Fassino et al. (2010)—Quant/P; Pham et al. (2015)—Quant/P +: Lewis et al. (2015)—Quant/P
	Reward dependance	–/0: Lewis et al. (2015)—Quant/P 0: Houeto et al. (2006)—Quant/P; Fassino et al. (2010)—Quant/P; Pham et al. (2015)—Quant/P
	Persistence	–: Pham et al. (2015)—Quant/P 0: Houeto et al. (2006)—Quant/P; Fassino et al. (2010)—Quant/P
	Self-directedness	0: Houeto et al. (2006)—Quant/P; Fassino et al. (2010)—Quant/P; Pham et al. (2015)—Quant/P
	Cooperativeness	0: Houeto et al. (2006)—Quant/P; Fassino et al. (2010)—Quant/P; Pham et al. (2015)—Quant/P
	Self-transcendence	–: Pham et al. (2015)—Quant/P 0: Houeto et al. (2006)—Quant/P 0/+: Fassino et al. (2010)—Quant/P
	Big five traits	
	Extraversion	–/0: Boel et al. (2016)—Quant/RCT
	Agreeableness	–/0: Boel et al. (2016)—Quant/RCT
	Conscientiousness	0: Boel et al. (2016)—Quant/RCT
	Neuroticism	0: Pham et al. (2015)—Quant/P; Boel et al. (2016)—Quant/RCT
	Openness	–/0: Boel et al. (2016)—Quant/RCT
	Affective/behavioral traits	
	Negative affective traits	–/+: Gilbert (2018)—Qual; Thomson et al. (2019)—Qual 0/+: Thomson et al. (2019)—Qual
	Positive affective traits	–/+: Gilbert (2018)—Qual; Thomson et al. (2020)—Qual
	Aggressiveness	0: Temel et al. (2006)—Quant/MA
	Anxiety	0: Castelli et al. (2006)—Quant/P
	Apathy	0: Temel et al. (2006)—Quant/MA; Appleby et al. (2007)—Quant/MA +: Denheyer et al. (2009)—Quant/Ret; Gilbert (2012)—Qual
	Hypersexuality	0: Temel et al. (2006)—Quant/MA; Appleby et al. (2007)—Quant/MA
	Hypomania	+: Lewis et al. (2015)—Quant/P
	Impulsivity	0: Lewis et al. (2015)—Quant/P 0/+: Pham et al. (2015)—Quant/P +: Hälbig et al. (2009)—Quant/CS
	Personality pathology	
	General personality pathology	0: Temel et al. (2006)—Quant/MA; Appleby et al. (2007)—Quant/MA; –/+ : Houeto et al. (2002)—Quant/Ret
	Personality disorders: obsessive-compulsive, paranoid	–: Castelli et al. (2006)—Quant/P
	Personality disorders: avoidant, dependent, passive-aggressive, self-frustrating, schizotypal, schizoid, histrionic, narcissistic, borderline, antisocial	0: Castelli et al. (2006)—Quant/P
Characteristic adaptations	Ability to pursue leisure activities	+: Törnqvist et al. (2007)—PVC/P; Kubu et al. (2017a,b)—PVC/P
	Ability to pursue social activities	+: Törnqvist et al. (2007)—PVC/P; Kubu et al. (2017a,b)—PVC/P
	Ability to pursue work activities	+: Törnqvist et al. (2007)—PVC/P; Kubu et al. (2017a,b)—PVC/P
	Difficulties with relationships	–/+: Gilbert (2018)—Qual +: Gilbert (2012)—Qual
	Difficulties with routine behaviors	–/+: Gilbert (2018)—Qual +: Gilbert (2012)—Qual
	Leisure/social/work goal importance	+/–: Kubu et al. (2018)—PVC/P
	Positive relationship-specific coping strategies	+: Baumann-Vogel et al. (2020)—Quant/CS
Narrative identity	Difficulties with self-image/self-estrangement	–/+: Gilbert et al. (2017)—Qual; Gilbert (2018)—Qual +: Gilbert (2012)—Qual

“–”evidence for negative association; “0”: no evidence for directional association; “+”: evidence for positive association; when –, 0, or + are separated by “/”, the study provided mixed evidence for associations [e.g., Lewis et al. (2015) provided evidence for negative and null associations between DBS and novelty-seeking]; References are followed by “–Method/Design”; “Quant” = quantitative methods; “Qual” = qualitative methods; “PVC” = patient valued characteristics (mixed methods); “MA” = meta-analysis; “Ret” = retrospective design; “CS” = Cross-sectional design; “P” = prospective design; “RCT” = randomized clinical trial.

The authors apologize for this error and state that this does not change the scientific conclusions of the article in any way. The original article has been updated.

## References

[B1] Appleby B. S. Duggan P. S. Regenberg A. Rabins P. V. (2007). Psychiatric and neuropsychiatric adverse events associated with deep brain stimulation: a meta-analysis of ten years' experience. Mov. Disord. Off. J. Mov. Disord. Soc. 22, 1722–1728. 10.1002/mds.21551 17721929

[B2] Baumann-Vogel H. Bodenmann G. Schmid J. Waldvogel D. Ineichen C. Baumann C. R. (2020). Partners' view after subthalamic deep brain stimulation: better relationships despite patients being less active. Clin. Parkinson. Relat. Disord. 3:100052. 10.1016/j.prdoa.2020.100052 34316635 PMC8298790

[B3] Boel J. A. Odekerken V. J. Geurtsen G. J. Schmand B. A. Cath D. C. Figee M.. (2016). Psychiatric and social outcome after deep brain stimulation for advanced Parkinson's disease. Mov. Disord. 31, 409–413. 10.1002/mds.26468 26660279

[B4] Castelli L. Perozzo P. Zibetti M. Crivelli B. Morabito U. Lanotte M.. (2006). Chronic deep brain stimulation of the subthalamic nucleus for Parkinson's disease: effects on cognition, mood, anxiety and personality traits. Eur. Neurol. 55, 136–144. 10.1159/000093213 16682797

[B5] Denheyer M. Kiss Z. H. Haffenden A. M. (2009). Behavioral effects of subthalamic deep brain stimulation in Parkinson's disease. Neuropsychologia 47, 3203–3209. 10.1016/j.neuropsychologia.2009.07.022 19664645

[B6] Fassino S. Daga G. A. Gramaglia C. Piero A. Zibetti M. Castelli L.. (2010). Novelty-Seeking in Parkinson's Disease after deep brain stimulation of the subthalamic nucleus: a case-control study. Psychosomatics 51, 62–67. 10.1016/S0033-3182(10)70660-5 20118442

[B7] Gilbert F. (2012). The burden of normality: from “chronically ill” to “symptom free.” New ethical challenges for deep brain stimulation postoperative treatment. J. Med. Ethics 38, 408–412. 10.1136/medethics-2011-100044 22431560

[B8] Gilbert F. (2018). Deep brain stimulation: inducing self-estrangement. Neuroethics 11, 157–165. 10.1007/s12152-017-9334-7

[B9] Gilbert F. Goddard E. Viaña J. N. M. Carter A. Horne M. (2017). I miss being me: phenomenological effects of deep brain stimulation. AJOB Neurosci. 8, 96–109. 10.1080/21507740.2017.1320319

[B10] Hälbig T. D. Tse W. Frisina P. G. Baker B. R. Hollander E. Shapiro H.. (2009). Subthalamic deep brain stimulation and impulse control in Parkinson's disease. Eur. J. Neurol. 16, 493–497. 10.1111/j.1468-1331.2008.02509.x 19236471

[B11] Houeto J.-L. Mallet L. Mesnage V. Tezenas du Montcel S. Béhar C. Gargiulo M.. (2006). Subthalamic stimulation in Parkinson Disease: behavior and social adaptation. JAMA Neurol. 63, 1090–1095. 10.1001/archneur.63.8.1090 16908734

[B12] Houeto J. L. Mesnage V. Mallet L. Pillon B. Gargiulo M. du Moncel S. T.. (2002). Behavioural disorders, Parkinson's disease and subthalamic stimulation. J. Neurol. Neurosurg. Psychiatry 72:701. 10.1136/jnnp.72.6.701 12023409 PMC1737905

[B13] Kubu C. S. Cooper S. E. Machado A. Frazier T. Vitek J. Ford P. J. (2017a). Insights gleaned by measuring patients' stated goals for DBS: more than tremor. Neurology 88, 124–130. 10.1212/WNL.0000000000003485 27913696 PMC5224715

[B14] Kubu C. S. Ford P. J. Lapin B. (2017b). Patients' Perceptions of Personality Change in Parkinson's Disease and Following Deep Brain Stimulation. Washington, DC: International Neuroethics Society meeting.

[B15] Kubu C. S. Frazier T. Cooper S. E. Machado A. Vitek J. Ford P. J. (2018). Patients' shifting goals for deep brain stimulation and informed consent. Neurology 91, e472–e478. 10.1212/WNL.0000000000005917 29959262 PMC6093764

[B16] Lewis C. J. Maier F. Horstkötter N. Zywczok A. Witt K. Eggers C.. (2015). Subjectively perceived personality and mood changes associated with subthalamic stimulation in patients with Parkinson's disease. Psychol. Med. 45, 73–85. 10.1017/S0033291714001081 25066623

[B17] Lhommée E. Boyer F. Wack M. Pélissier P. Klinger H. Schmitt E.. (2017). Personality, dopamine, and Parkinson's disease: insights from subthalamic stimulation. Mov. Disord. 32, 1191–1200. 10.1002/mds.27065 28643887

[B18] Pham U. Solbakk A.-K. Skogseid I.-M. Toft M. Pripp A. H. Konglund A. E.. (2015). Personality changes after deep brain stimulation in Parkinson's Disease. Parkinson's Dis. 7:490507. 10.1155/2015/490507 25705545 PMC4325225

[B19] Temel Y. Kessels A. Tan S. Topdag A. Boon P. Visser-Vandewalle V. (2006). Behavioural changes after bilateral subthalamic stimulation in advanced Parkinson disease: a systematic review. Parkinson. Relat. Disord. 12, 265–272. 10.1016/j.parkreldis.2006.01.004 16621661

[B20] Thomson C. J. Segrave R. A. Carter A. (2019). Changes in personality associated with deep brain stimulation: a qualitative evaluation of clinician perspectives. Neuroethics 2, 1–16. 10.1007/s12152-019-09419-2

[B21] Thomson C. J. Segrave R. A. Racine E. Warren N. Thyagarajan D. Carter A. (2020). “He's back so I'm not alone”: the impact of deep brain stimulation on personality, self, and relationships in Parkinson's Disease. Qualitat. Health Res. 2020:1049732320951144. 10.1177/1049732320951144 32856559

[B22] Törnqvist A. Ahlström G. Widner H. Rehncrona S. (2007). Fulfilment of patients' goals after thalamic deep brain stimulation: a follow-up study. Parkinson. Relat. Disord. 13, 29–34. 10.1016/j.parkreldis.2006.06.005 16928465

